# Specific knockdown of uPA/uPAR attenuates invasion in glioblastoma cells and xenografts by inhibition of cleavage and trafficking of Notch -1 receptor

**DOI:** 10.1186/1476-4598-10-130

**Published:** 2011-10-17

**Authors:** Hari Raghu, Christopher S Gondi, Dzung H Dinh, Meena Gujrati, Jasti S Rao

**Affiliations:** 1 Department of Cancer Biology and Pharmacology, University of Illinois College of Medicine, One Illini Drive, Peoria, IL 61605, USA; 2 Department of Neurosurgery, University of Illinois College of Medicine, One Illini Drive, Peoria, IL 61605, USA; 3 Department of Pathology, University of Illinois College of Medicine, One Illini Drive, Peoria, IL 61605, USA

**Keywords:** uPA/uPAR, invasion, glioblastoma, Notch 1 receptor

## Abstract

**Background:**

uPA/uPAR is a multifunctional system that is over expressed in many cancers and plays a critical role in glioblastoma (GBM) invasion. Previous studies from our lab have also shown that uPA/uPAR down regulation inhibits cancer cell invasion in SNB 19 GBM cells.

**Methods:**

As Notch 1 is known to be over expressed and promotes invasion in glioblastoma, we therefore tested our hypothesis of whether down regulation of uPA/uPAR, singly or in tandem, attenuates GBM invasion via Notch 1 receptor. Targeted down regulation of uPA/uPAR, either singly or simultaneously, inhibited the anchorage independent growth of U251MG and GBM xenograft cell lines 4910 and 5310 as assessed by soft agar colony formation assay. Expression of all four Notch receptors was confirmed in GBM tissue array analysis by immunohistochemistry.

**Results:**

Down regulation of uPA/uPAR, either singly or simultaneously, in U251 MG and tumor xenografts inhibited the cleavage of the Notch receptor between the Gly 1743 and Val 1744 positions, thereby suggesting inhibition of activated cytosolic fragment-related Notch gene transcription. Morphological analysis confirmed inhibition of NICD when U251 MG cells were treated with puPA, puPAR or pU2. uPA/uPAR down regulation inhibited Notch 1 mRNA in all three examined cell lines. uPA/uPAR shRNA down regulated nuclear activation of NF-κB subunits and phosphorylation of AKT/mTOR pathway in U251 MG and GBM xenografts. puPA down regulated NICD and HES induced phosphorylation of AKT/ERK and NF-κB. Down regulation of Notch 1 using siRNA inhibited uPA activity as shown by fibrinogen zymography. It also decreased uPA expression levels as shown by western blotting. Exogenous addition of uPA activated Notch 1 in uPAR antisense U251 MG cells and also in uPAR antisense cells transfected with siRNA against Delta and Jagged. The Notch 1 receptor co-localized with LAMP-1, a marker for lysosomes in uPA, uPAR and U2, down regulated U251 MG cells which probably indicates inhibition of Notch 1 receptor trafficking in GBM cells. Notch 1 expression was significantly inhibited in puPA- and pU2-treated pre-established intracranial tumors in mice.

**Conclusions:**

Overall our results show that down regulation of uPA/uPAR, either singly or simultaneously, could be an effective approach to attenuate Notch 1 receptor cleavage, signaling and endosomal trafficking in U251MG cells and xenografts, and ultimately inhibiting GBM invasion.

## Background

In spite of the recent development of targeted therapies (e.g., Temozolomide and avastin), glioblastoma (GBM) still continues to be a medical challenge. Identification of novel targets such as uPA and uPAR that are over expressed in glioblastoma and understanding the different signaling pathways they regulate will greatly enhance the clinical treatment and outcome for patients affected with glioblastoma.

The uPA/uPAR system is a multifunctional system which is involved in several cellular processes like migration, angiogenesis and invasion. Studies by others and us have conclusively shown that the uPA/uPAR system significantly correlates to tumor aggressiveness and poor outcome. Our studies have also shown that shRNA constructs directed against uPA/uPAR, either singly or in combination, have a significant inhibitory effect on the migration, invasion and angiogenesis of GBM cells and xenografts.

Notch signaling is a highly conserved pathway playing an important role during embryo development and adulthood. In mammals, it consists of four receptors, namely Notch 1, Notch 2, Notch 3 and Notch 4 [1,2]. The Notch receptor is a hetero-oligomer and a single-pass transmembrane receptor. The transmembrane monomer is bound through non-covalent interactions to a fully extracellular monomer. Notch is involved in cell proliferation, apoptosis, differentiation, survival and stabilization of arterial endothelial fate, angiogenesis and many other functions [3,4]. Notch signaling is deregulated in many cancers including glioblastoma [5]. The Notch receptor contains the extracellular peptide which contains EGF repeats that non-covalently associate with the transmembrane peptide and binds the Notch ligands (e.g. Delta and Jagged). Upon ligand binding, the Notch receptor is cleaved and sensitized by ADAM metalloproteases and gamma secretase. This leads to cleavage of NICD (Notch intracellular domain) to the nucleus, and the NICD forms a complex with CBF-1 and the transcriptional co-activator MAML for transcription of Notch genes [6]. Previous studies have shown that inhibition of Notch signaling by pharmacological or genetic means leads to cell cycle arrest and suppression of cell growth [7-9].

We have shown that uPA and uPAR downregulation inhibits invasion in SNB19 glioma cells by decreasing phosphorylation of the Ras-activated FAK, p38MAPK, JNK and ERK1/2, as well as the MEK-activated phosphatidylinositol 3-kinase, AKT and mTOR pathway, indicative of a feedback signaling mechanism of the uPAR/uPA system [10]. Moreover, studies have shown that Notch 1 signaling is known to cross talk with ERK, NF-κB and with the PI3-K/AKT/mTOR pathway [1]. Notch has multiple roles in invasion and angiogenesis of many human cancers but the mechanisms are not understood [11,12]. In this study, we have shown that down regulation of uPA and uPAR inhibits invasion of U251 MG cells and xenograft glioma cells by inhibition of Notch- related gene transcription, signaling mechanism and targeting Notch 1 to the lysosomal pathway. We have also shown that uPA/uPAR down regulation inhibits Notch 1 expression in pre-established tumors in nude mice. Overall, our results suggest that down regulation of uPA/uPAR, either singly or in combination, results in the inhibition of glioma cell invasion via inhibition of Notch 1 receptor cleavage, signaling and endosomal trafficking of the Notch 1 receptor.

## Methods

### Cells and Reagents

U251 MG cells were obtained from American Type Culture Collection, (Manassas, VA), and glioma xenograft cell lines (5310 & 4910) were kindly provided by Dr. David James (University of California, San Francisco, San Francisco, CA). All cell lines were cultured as described previously [10]. Antibodies to Notch 1, 2, 3 and 4 were obtained from Santa Cruz Biotechnology (Santa Cruz, CA). Antibodies to cleaved Notch between Gly 1743 and Val 1744 were obtained from Cell Signaling (Beverly MA). Antibodies to extracellular domain of Notch were obtained from Abcam (Cambridge, UK). Antibodies to Delta and Jagged were obtained from Santa Cruz Biotechnology, (Santa Cruz, CA). Antibodies to RBPJκ, Lamin B, alpha tubulin, pAKT, NF-κB p65, MEK, pERK, pmTOR Ser 2448, were obtained from Cell Signaling (Beverly MA). Antibodies to uPA and uPAR were purchased from R&D Systems (Minneapolis, MN). Notch 1 siRNA and antibodies to GAPDH and LAMP-1 were purchased from SCBT (Santa Cruz, CA).

### uPA and uPAR shRNA constructs

shRNA sequences targeting uPAR and uPA were constructed according to our previous publication [13].

### Transfection with shRNA constructs

1.5 × 10^5 ^cells were plated in 100 mm petri dishes for each transfection experiment. The cells were transfected in serum-free L-15 media using 10 μg of Fugene reagent (Roche, USA) according to the manufacturer's instructions. The following constructs were used for transfection: puPA, puPAR, pU2 (both uPA and uPAR) and pSV (scrambled vector). No plasmid was introduced in the control plates. After 12 hrs of transfection, the serum-free media was replaced with serum-containing media and the cells were left in the incubator at 37°C for 48 hrs. The media was then replaced with serum-free media, and conditioned media was collected 12 hrs later. Cells were harvested for isolation of total RNA and/or total cell lysates. Conditioned media was used for fibrinogen plasminogen zymography.

### Fibrinogen plasminogen zymography

We used fibrin zymography to determine the activity of the plasminogen activators as previously described [14]. The samples were subjected to SDS-PAGE with 10% gels that contained fibrinogen and plasminogen. Following electrophoresis, the gels were washed twice with 2.5% (v/v) Triton X-100 for 30 min each time to remove SDS. Finally, the gels were incubated with 0.1 M glycine buffer (pH 7.5) at 37°C overnight, stained with amido black, and then destained. The final gel had a uniform background except in regions to which uPA had migrated and cleaved its substrate.

### Western blotting

U251 MG, 5310 and 4910 cells were left untreated or transfected with puPA, puPAR or pU2. Cells were collected and whole cell lysates were prepared by lysing cells in RIPA lysis buffer containing a protease inhibitor cocktail (Sigma, St. Louis, MO). Equal amounts of protein fractions, immunoprecipitates or lysates were resolved by SDS-PAGE and transferred to a polyvinylidene difluoride membrane. Proteins were detected with appropriate primary antibodies followed by HRP-conjugated secondary antibodies. Comparable loading of proteins was verified by reprobing the blots with an antibody specific for the housekeeping gene product, GAPDH.

### Reverse transcriptase polymerase chain reaction

Total RNA was isolated from U251MG, 4910 and 5310 cells using the TRIzol reagent as per the standard protocol. Total RNA was treated with DNAse I (Invitrogen, Carlsbad, CA) to remove contaminating genomic DNA. PCR analysis was done using the one-step reverse transcription-PCR kit (Invitrogen, Carlsbad, CA). Glyceraldehyde 3-phosphate dehydrogenase (GAPDH) was used as an internal control. The following primers were used:

Notch 1 Forward 5'-CAGCTTGCACAACCAGACAGAC-3'

Notch 1 Reverse 5'-ACGGAGTACGGCCCATGTT-3'

Notch 2 Forward AACTGTCAGACCCTGGTGAAC

Notch 2 Reverse CGACAAGTGTAGCCTCCAATC

Notch 3 Forward TGACCGTACTGGCGAGACT

Notch 3 reverse CCGCTTGGCTGCATCAG

Notch 4 Forward AGTCCAGGCCTTGCCAGAACG

Notch 4 Reverse GTAGAAGGCATTGGCCAGAGAG

GAPDH:5'-CGGAGTCAACGGATTTGGTCGTAT-3'sense

5'-AGCCTTCTCCATGGTGGTGAAGAC-3' antisense

The PCR conditions were as follows: 95°C for 5 min., followed by 30 cycles of 95°C for 1 min., and annealing temperature set according to the AT and GC content of the primers.

### NF-κB family transcription factor assay

Nuclear extracts from untreated U251 MG, 5310 and 4910 cells transfected with pSV, puPA, puPAR or pU2 were assayed for activation of NF-κB transcription factor by TransAM NF-κB family activation kit (Active Motif, Carlsbad, CA) according to the manufacturer's instructions.

### Immunofluorescence assay

U251 MG, 4910 and 5310 cells were either transfected with pSV, puPA, puPAR or pU2 or left untransfected. The cells were then fixed, permeabilized, stained with primary antibodies against Notch 1 and LAMP-1, washed, incubated with Alexa fluor tagged secondary antibodies, washed with PBS and mounted in an anti-fading reagent containing DAPI. The cells were then visualized using a confocal microscope using Olympus Fluoview software.

### uPA/uPAR quantification by ELISA

Conditioned medium from mock and pSV, puPA, puPAR, or pU2-transfected U251 MG, 5310 and 4910 cells were subjected to ELISA for uPA and uPAR (R&D Systems, Minneapolis, MN) according to the manufacturer's instructions.

### Soft colony agar assay

Anchorage independent growth was determined by assaying colony formation in soft agar [15]. Briefly, U251 MG, 5310 and 4910 cells which were either untreated or transfected with pSV, puPA, puPAR or pU2 were suspended in DMEM or RPMI containing 10% FBS and 0.33% sea plaque low-melting temp agarose. 2 ml containing 2 × 10^4 ^cells were plated on a 35 mm dish over a 3 ml layer of solidified DMEM/RPMI 10% FBS and 0.6% agarose. The cells were fed by adding 200 μl of RPMI/10% FBS or with DMEM/10% FBS. The colonies were photographed at 10x and 20x magnification after 4 weeks.

### Matrigel invasion assay

Invasion of U251MG and 4910 cells was determined by a Matrigel invasion assay as described previously [16]. Briefly, U251MG, 4910 cells which were either left untreated, treated with DAPT (N-[N-(3,5-Difluorophenacetyl)-L-alanyl]-S-phenylglycine t-butyl ester ( NICD inhibitor) for 1 h at 37°C or transfected with pSV, puPA, puPAR or pU2 were detached, washed twice in PBS, suspended in serum-free medium and seeded in the upper chamber of a Transwell insert (8-μm pores) coated with matrigel (1 mg ml^-1^) (Collaborative Research Inc., Boston, MA). The lower chamber was filled with 700 μl of complete medium. After 18 hrs of incubation period, the non-migrated cells in the upper chamber were gently scraped away, and invaded cells present on the lower surface of the insert were stained with Hema-3 (Fisher Diagnostics, Middletown, VA). Photographs of the invaded cells were taken with a light microscope (Olympus IX-71, Minneapolis, MN).

### Co-localization of Notch 1 with LAMP-1

Semi-confluent U251 MG cells grown in 8-well chamber slides were left untreated or transfected with pSV,puPA, puPAR and pU2. After 48 hrs, the cells were washed, fixed with 2% paraformaldehyde, permeabilized in 0.5% Triton X-100, and stained with primary antibodies against Notch-1 and LAMP-1. The cells were then washed and further incubated with appropriate secondary Alexa fluor antibodies, and examined under an Olympus Fluoview microscope.

### Animal experiments

U251 MG cells (2 × 10^6^) were injected into the brains of nude mice using a stereotactic frame. After 8-10 days the mice were treated with pSV, puPAR, puPA and pU2. The *in vivo *intracranial delivery of the vectors was performed using Alzet mini-osmotic pumps (Direct Corp, Cupertion, CA) at the rate of 0.25 μl/h [mock (PBS), 150 μg of Vector DNA, 150 μg of puPA, 150 μg of puPAR and 150 μg of pU2]. All experiments were performed in compliance with institutional guidelines established by the University Of Illinois College Of Medicine at Peoria. After five weeks or when the control mice started showing symptoms, mice were euthanized by cardiac perfusion with formaldehyde. The brains were then removed and paraffin embedded following standard protocols. Sections were prepared with H&E. In each case sections were blindly reviewed and scored semi-quantitatively for tumor size. Sections were observed and immunohistochemical analysis for Notch 1 was done on paraffin-embedded brain tumor sections of mice treated with pSV, puPA and pU2 with specific antibody for Notch 1.

## Results and Discussion

### Notch receptors are expressed in human glioblastoma tissue microarray and in glioblastoma cells and glioma xenograft cell lines

**Figure 1:**
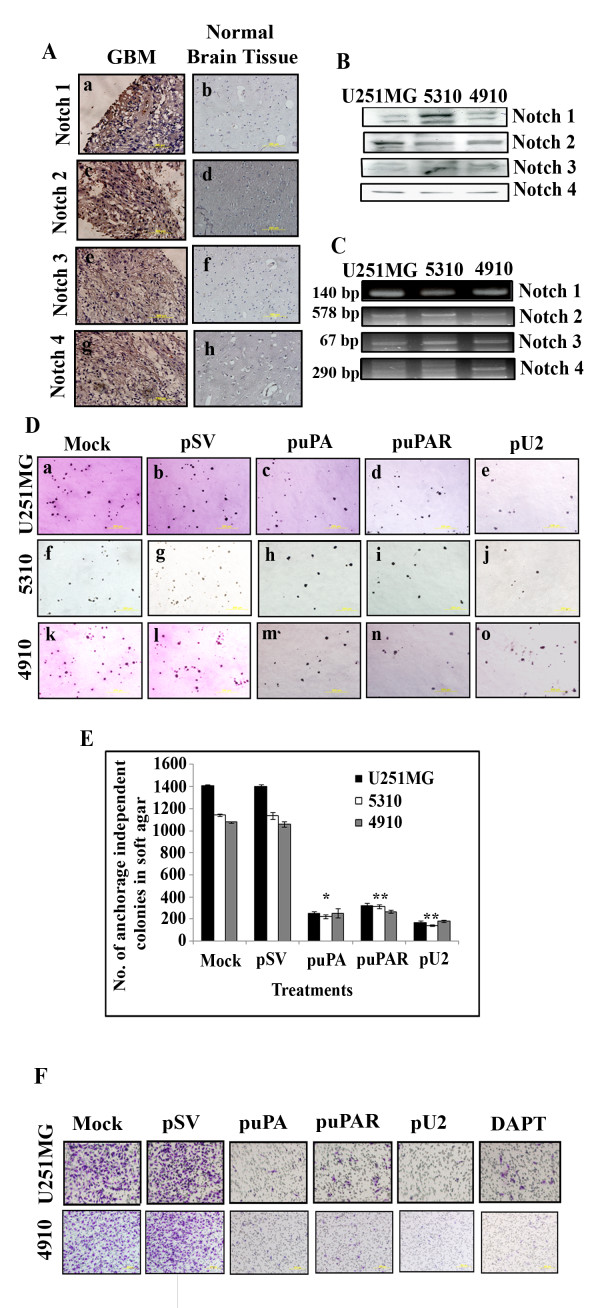
**Expression of Notch signaling components in glioblastoma cancer cell line and xenograft cell lines**. (A) Representative composite of Notch 1, 2, 3 and 4 receptors by immunohistochemistry in glioblastoma human tissue array. (B) Western blot analysis of Notch signaling components in three different cells, namely U251 MG and xenograft cell lines 5310 and 4910. (C) RT-PCR analysis of Notch 1, 2, 3 and 4 in U251 MG and xenograft cell lines, 5310 and 4910. (D) shRNA against uPA, uPAR and U2 inhibits anchorage independent growth of U251 MG and glioblastoma xenograft cell lines in soft agar. (Panels a to e - U251 MG, panels f to j - 5310 and panels k to o - 4910). A total of 1 × 10^5 ^cells from each cell line were left either untransfected or transfected with pSV, puPA, puPAR and pU2, and these cells were then plated in soft agar. Representative photographs are shown which were taken at four weeks after the colonies were stained with crystal violet. (E) Quantitative representation of number of anchorage-independent colonies in soft agar colony formation assay before and after transfection with pSV, puPA, puPAR and pU2. Values are mean ± S.D. of three independent experiments, in comparison with controls (* P < 0.01, ** P < 0.001) (F) shRNA against uPA, uPAR and U2 inhibits GBM invasion by a matrigel invasion assay as described previously in Material and Methods.

To determine if Notch is expressed in glioblastoma tissues and cells, we wanted to check for Notch receptor expression by GBM human tissue array, western blot and by RT-PCR in U251 MG, 4910 and 5310 cells. Consistent with previous reports, Notch 1, Notch 2, Notch 3 and Notch 4 expression by immunohistochemistry was observed in the GBM tissue array (Figure 1A, panels a, c, e, and g ). Notch receptors 1 to 4 were minimally expressed in normal brain tissue. (Figure 1A, panels b, d, f, h) Further confirmation of our GBM tissue array results was accomplished with immunofluorescent analysis (data not shown). Lysates from all the three cell lines showed expression of Notch 1, 2, 3, and 4 (Figure 1B) and results of mRNA analysis by RT-PCR also showed gene expression of all the four receptors (Figure 1C).

### shRNA-mediated uPA/uPAR downregulation inhibits anchorage independent growth of U251 MG, 5310 and 4910 cells

U251 MG, 5310, 4910 cells were left untransfected or transfected with puPA, puPAR and pU2 were analyzed by a colony formation assay in soft agar (Figure 1D). puPA-, puPAR- and pU2-transfected U251 MG (Figure 1D, panel a to e), 5310 (Figure 1D, panel f to j) and 4910 (Figure 1D, panel k to o) cells showed significant decreases in colony number (**p < 0.0001). Quantitative analysis of the number of anchorage independent colonies in soft agar after shRNA transfection in U251 MG, 5310 and 4910 cells is shown in Figure 1E. The colony size was also decreased in the shRNA-transfected cells in comparison to the controls. A complete recovery in both colony number and size was obtained when exogenous uPA and ruPAR was added (data not shown) indicating a specific role for uPA and uPAR in anchorage-independent growth of U251, 5310 and 4910 cells. To further confirm our results, we performed a matrigel invasion assay in U251MG and 4910 cells left untransfected or transfected with SV, puPA, puPAR and pU2 and with DAPT (an NICD inhibitor). Results showed that invasion was inhibited in puPA, puPAR, pU2 and DAPT-treated conditions compared with controls, suggesting that inhibition of invasion by shRNA constructs against uPA, uPAR and U2 is mediated by Notch-1 receptor (Figure 1F).

### puPA, puPAR and pU2 inhibit cleavage of Notch receptor in U251MG and xenograft cell lines

**Figure 2:**
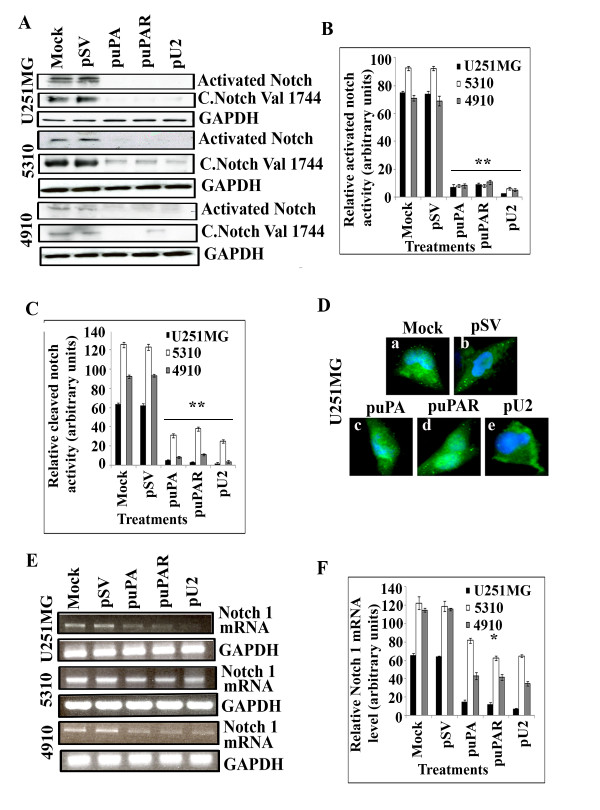
**Transcriptional inactivation of uPA and/or uPAR by shRNA inhibits cleavage of Notch 1 receptor, Notch 1 gene expression in U251 MG, 4910 and 5310 xenograft cells**. (A) Whole cell lysates were prepared from U251 MG, 4910 and 5310 cells which were left untransfected, or transfected with pSV, puPA, puPAR and pU2 to evaluate the protein levels of activated Notch 1 and for Notch 1 cleavage at the Val 1744 position. Blots were probed with GAPDH antibody to analyze for equal loading of proteins. (B) & (C) Quantitative analysis of relative Notch 1 expression and cleaved Notch expression in U251 MG, 5310 and 4910 cells. (D) Immunofluorescence analysis of U251MG cells left untransfected or transfected with pSV, puPA, puPAR and pU2 and stained for NICD (Notch intracellular domain) and images taken with Olympus Fluoview microscope. (E) RT-PCR analysis of Notch 1 mRNA expression in (a) U251 MG, 5310 and 4910 cells which were left untransfected or transfected with pSV, puPA, puPAR and pU2. GAPDH was used as an internal control. (F) Quantitative representation of Notch 1 mRNA levels in U251MG, 5310 and 4910 cells. Values are mean ± S.D. of three independent experiments (* P < 0.01, **P < 0.001).

Notch receptor is cleaved by four enzymes and each cleaved product is significant to its function. However, cleavage of Notch 1 by gamma secretase is very critical for the function of the Notch receptor. Cleavage of Notch 1 by γ secreatase results in the release of NICD (Notch intracellular domain), and release of NICD to the nucleus is critical for Notch gene expression. In all of the three cell lines examined, we observed that puPA, puPAR and pU2 inhibited the cleavage of the Notch receptor as indicated by the absence of a cleaved band at 80 Kda compared with the controls (Figure 2A). Notch 1 is a transmembrane domain protein that is involved in the development and determination of cell fate [17]. During maturation, the Notch molecule is cleaved by a furin-like convertase at its extracellular domain [18]. Upon ligand binding, the carboxy terminal Notch 1 fragment is released and is further cleaved at Gly 1743 and Val 1744 [19,20]. Endogenous levels of cytosolic domain are observed only upon cleavage between Gly 1743 and Val 1744. Results show cleavage of Notch at the Val 1744 position is inhibited in puPA- and pU2-transfected U251, 4910 and 5310 cells (Figure 2A). Quantitative representation of activated Notch 1 and cleaved Notch 1 levels is shown in Figure 2B and Figure 2C, respectively. To further confirm our western blotting results, we checked for the NICD domain in U251 MG cells by staining the cells with an antibody to NICD in untreated U251 MG cells and in U251 MG cells transfected with pSV, puPA, puPAR and pU2. As demonstrated by our western blot results, we observed increased expression of NICD in mock and pSV-treated cells (Figure 2D, panel a & b) while the cells treated with the three shRNA constructs showed very little staining (Figure 2D, panel c to e). NICD staining was seen as small green spots at the periphery and at the center of the cells.

### puPA, puPAR and pU2 inhibits the gene expression of Notch 1 mRNA in all the three cell lines examined

To determine whether puPA, puPAR and pU2 inhibit Notch 1 mRNA, we subjected controls and shRNA-transfected U251 MG, 5310 and 4910 cells to RT PCR for Notch 1 gene expression. Results showed that simultaneous down regulation of uPA and uPAR by pU2 efficiently down regulated Notch 1 mRNA in all three cell lines compared with puPA and puPAR (Figure 2E). Quantitative analysis of the RT-PCR results is shown in Figure 2F. These results suggest that simultaneous down regulation of both uPA and uPAR with pU2 is a better approach to knockdown Notch 1 mRNA than using single shRNA constructs.

### Notch inhibits uPA expression and activity in glioblastoma cells and xenografts

**Figure 3:**
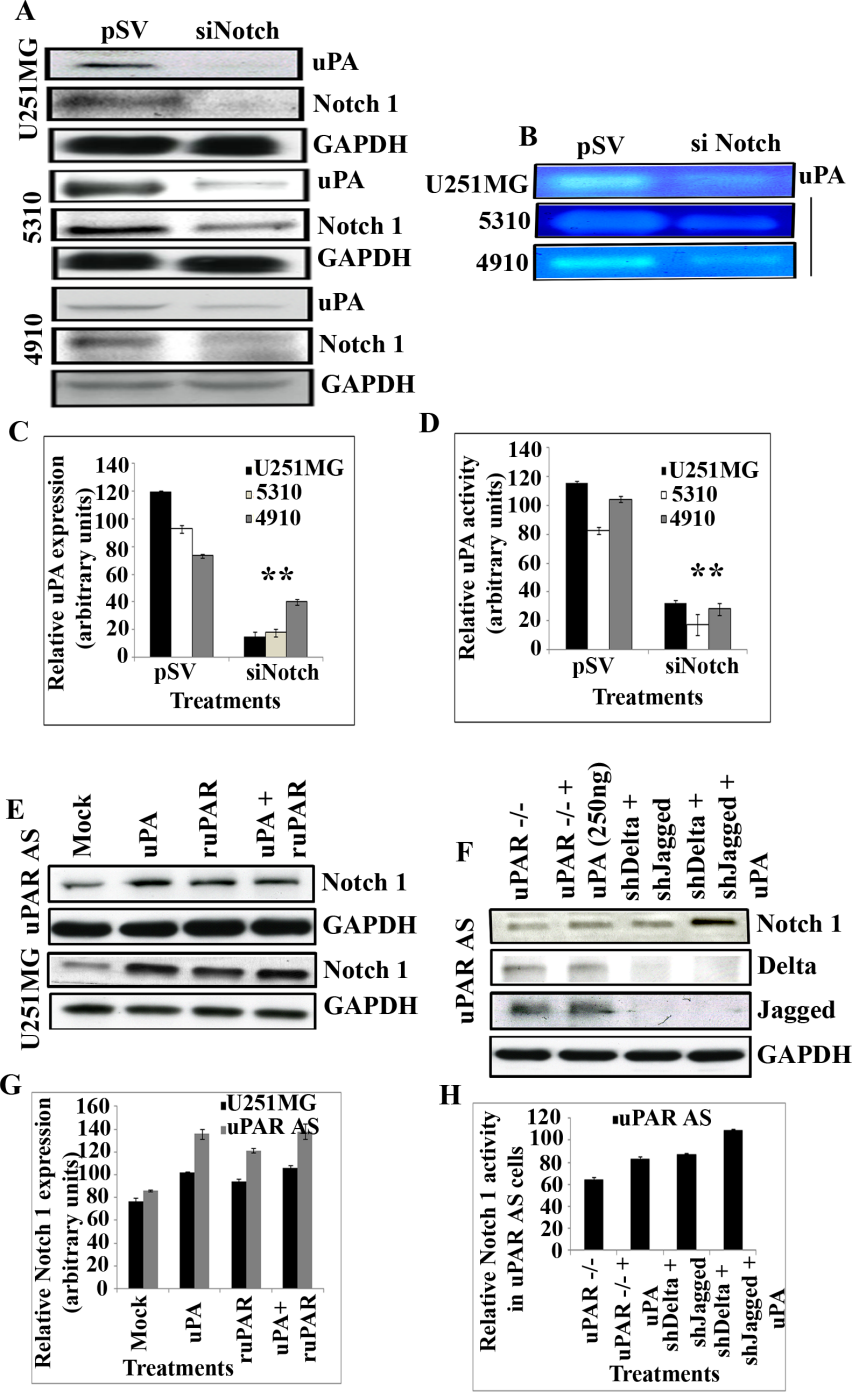
**uPA positively regulates Notch in glioblastoma cells**. (A) U251 MG, 5310 and 4910 cells were transfected with pSV (scrambled Vector) or with siRNA against Notch 1 and analyzed for uPA and Notch 1 expression by western blotting. Blots were probed with GAPDH antibody to analyze for equal loading of proteins. (B) Fibrin zymography of U251 MG, 5310 and 4910 cells transfected with pSV (scrambled Vector) and siNotch 1 was performed to analyze for uPA activity. (C) & (D) Quantitative expression of relative uPA expression and uPA activity in pSV and siNotch-transfected cells. (E) Exogenous addition of purified uPA activates Notch 1 receptor in uPAR antisense cells and in U251 MG cells. Whole cell lysates prepared from uPAR antisense and U251 MG cells, which were treated with purified uPA (250 ng), ruPAR (50 ng) and in combination, were checked for Notch 1 expression by Western blotting analysis. Blots were probed with GAPDH antibody to analyze for equal loading of lysates. (F) Whole cell lysates prepared from uPAR antisense cells which were left untreated, treated with purified uPA, transfected with shRNA against Delta and Jagged (Notch 1 ligands) with and without stimulation with purified uPA and probed for Notch 1, Delta and Jagged by Western blotting. Blots were probed with GAPDH antibody to analyze for equal loading of lysates. (G) Quantitative analysis of relative Notch 1 expression represented in (E) and (H) Quantitative analysis of relative Notch 1 expression represented in (F). Values are mean ± S.D. of three independent experiments in comparison with the controls (* P < 0/01, **P < 0.001).

To ascertain whether Notch plays a role in regulating uPA activity and expression, we transfected U251 MG, 5310 and 4910 cells with scrambled vector siRNA and siRNA to Notch 1 and checked for uPA activity by fibrinogen zymography and uPA expression using western blotting. In all three cell lines tested, results showed that Notch siRNA downregulated uPA expression (Figure 3A) and uPA activity (Figure 3B). This probably suggests that Notch and uPA regulate each other through a positive feedback mechanism. Quantitative expression of relative uPA expression and relative uPA activity is shown in Figures 3C and Figure 3D, respectively.

### Exogenous addition of uPA and uPAR activate Notch 1 in uPAR antisense and in U251 MG cells

Our previous results showed that shRNA against uPA and uPAR inhibits Notch expression and cleavage (Figure 2A). We wanted to check if addition of exogenous uPA and uPAR activates the Notch 1 receptor in uPAR antisense cells and in U251 MG cells. Our results indicate that upon exogenous addition of uPA and ruPAR, individually and in combination, Notch 1 expression was activated in uPAR antisense cells and in U251 MG cells (Figure 3E). This probably suggests that uPA independent of its role as a uPAR ligand can activate the Notch 1 receptor in glioblastoma cells.

### uPA activates Notch 1 receptor independent of Notch ligands, Delta and Jagged

To further clarify the role of uPA as a ligand for Notch 1 in glioblastoma cells, we wanted to ascertain if uPA activates Notch 1 in uPAR antisense cells in both the presence and absence of Notch ligands, namely Delta and Jagged. For this purpose, we used siDelta and siJagged transfected uPAR antisense cells and stimulated these cells with uPA. Surprisingly, we found that in uPAR antisense cells, exogenous uPA activated Notch 1 and showed a further increase in fold activation in the presence of siDelta and Jagged siRNA (Figure 3F). These results suggest that Notch 1 can be activated by uPA by independent of uPAR and Notch ligands, Delta and Jagged. Quantitative expression of Notch 1 expression from Figure 3E &3F is depicted in Figure 3G and Figure 3H, respectively. Overall, these results indicate that Notch 1 and uPA regulate each other in glioblastoma and GBM uPAR antisense cells.

### puPA, puPAR and pU2 inhibits NICD and HES-induced AKT, NF-κB and ERK pathways in U251 MG and 4910 cells

**Figure 4:**
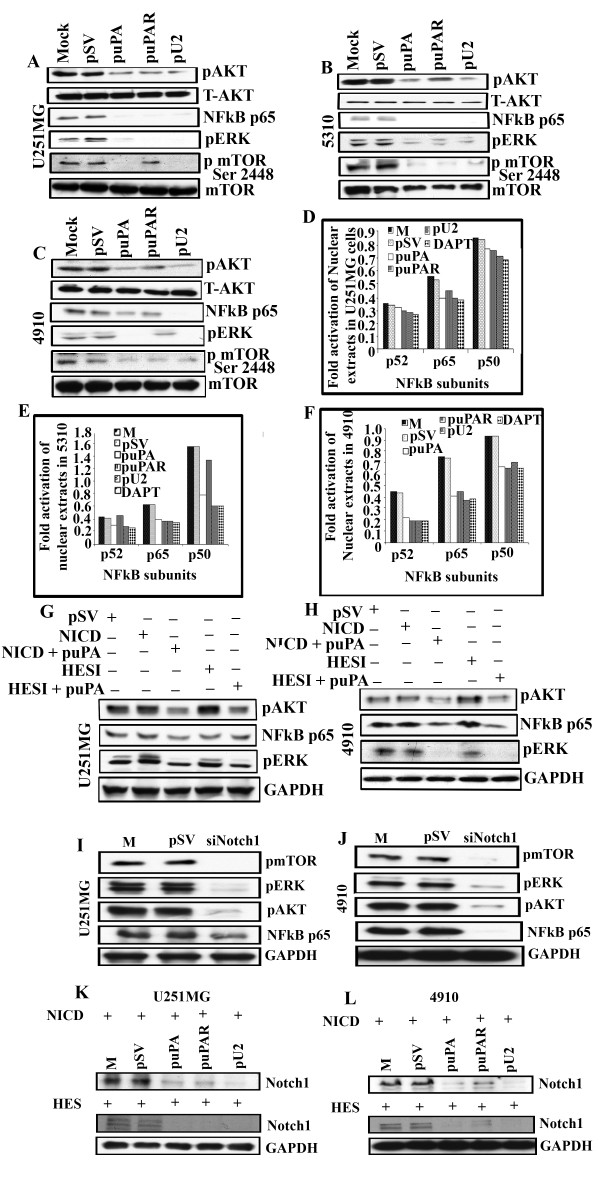
**puPA inhibits NICD and HES1-induced AKT, NF-κB and ERK phosphorylation in glioblastoma cells**. Down regulation of uPA/uPAR inhibits AKT, NF-κB, ERK and mTOR pathway in (A) U251 MG (B) 5310 and (C) 4910 cells. pSV: scrambled vector; puPA: shRNA against uPA; puPAR: shRNA against uPAR and pU2: shRNA against both uPA and uPAR, DAPT-GSI (Gamma secretase inhibitor). puPA, puPAR and pU2 inhibits nuclear activation of p52, p65 and p50 in (D) U251 MG (E) 5310 and (F) 4910 cells. Nuclear extracts prepared from U251 MG, 5310 and 4910 cells which were left untransfected, transfected with pSV, puPA, puPAR, and pU2 and treated with DAPT and analyzed for nuclear activation of p52, p50 and p65 subunits of NF-κB using the Active Motif Transcription factor analysis kit following the manufacturer's instructions. puPA inhibits NICD and HES- induced AKT and ERK phosphorylation and NF- p65 in U251 MG and 4910 cells. Whole cell lysates from (G) U251 MG or from (H) 4910 xenograft cells over expressing NICD (Notch intracellular domain) or HES were transfected with pSV, transfected with puPA and were immunoblotted for pAKT, NF-κB p65 and pERK. Blots were reprobed with GAPDH to analyze for equal loading of proteins. Data represents average of triplicates normalized to GAPDH (**p < 0.01). (I & J) siRNA against Notch 1 inhibits phosphorylated forms of mTOR/ERK/AKT and NF- κB p65 in (I) U251MG and (J) 4910 cells. Whole cell lysates from U251MG and 4910 cells were left untreated, transfected with pSV and siNotch for 48 hrs and were immunoblotted for pmTOR, pERK, pAKT and NF- κB p65. A representative blot of three independent experiments is shown. Blots were reprobed with e GAPDH to analyze for equal loading of proteins. (K&L) shRNA against uPA, uPAR and U2 inhibited NICD/HES1 induced Notch 1 expression in U251MG and 4910 cells. (K) Whole cell lysates from (G) U251 MG or from (H) 4910 xenograft cells over expressing NICD (Notch intracellular domain) or HES were transfected with pSV, puPA, puPAR and pU2 and were immunoblotted for Notch 1 expression. A representative blot of three independent experiments is shown. Blots were reprobed with GAPDH to analyze for equal loading of proteins.

Previously, we have shown that down regulation of uPA, uPAR and pU2 in SNB19 cells leads to decreased phosphorylation of AKT and mTOR at the Ser 2448 position, and ERK downstream of AKT. To further confirm these results in the cells lines used in this study, we checked for phosphorylation of AKT, ERK and mTOR after transfection with shRNA against uPA/uPAR, singly and in combination. As anticipated, we observed that transfection with puPA-, puPAR- and pU2-transfected cells significantly down regulated phosphorylation of AKT, mTOR and ERK pathways in the three cell lines used in this study. (Figure 4A-C). Previous studies have shown that NF-κB is a downstream target for ERK [21]. To confirm whether uPA/uPAR downregulation inhibits NF-κB, which is downstream to ERK, we checked the nuclear activation of p50 (NF-κB1), p52 (NF-κB2) and p65 (Rel A). As expected, we observed significant reduction in the nuclear activation of p50, p52 and p65 in all three cell lines tested (Figure 4D-F). Overall, these results suggest that shRNA against uPA, uPAR and U2 inhibits AKT/mTOR, ERK and NF-κB pathways in glioblastoma cells and xenografts.

The cross talk between Notch signaling and AKT/mTOR pathways has been reported in the literature in many studies [22-24]. Previous reports in the literature [25,26] have shown that there is a cross talk between Notch and NF-κB. To determine whether puPA down regulates Notch-induced AKT, NF-κB and ERK pathways, we overexpressed NICD and HES1 in U251 MG and 4910 cells. We also transfected these cells with puPA to check if puPA inhibits NICD and HES-induced phosphorylation of AKT, NF-κB and phosphorylation of ERK. Interestingly, we observed activation of AKT, ERK and NF-κB in pSV-transfected cells and in NICD and HES1-overexpressed cells (Figure 4G and 4H). AKT, ERK phosphorylation and NF-kB p65 levels were significantly down regulated upon transfection with puPA (Figure 4G and 4H). To confirm the cross talk of AKT/mTOR/NF- κB/ERK pathways with Notch-1, U251MG and 4910 cells were left untreated or treated with pSV and siNotch 1 and checked for the expression of pmTOR, pERK, pAKT and NF-kB p65. Results showed that siNotch 1 inhibited phosphorylation of AKT, ERK, mTOR and NF-kB levels compared with controls in both the cell lines tested (Figure 4I and Figure 4J). To further confirm if NICD/HES1-overexpressed Notch 1 is inhibited by puPA, puPAR and pU2 shRNA constructs, we checked for Notch 1 expression in U251MG and 4910 cells after transfection with shRNA constructs against uPA, uPAR and U2. Results showed that NICD/HES1 induced Notch1 expression levels were significantly reduced in uPA andU2 transfected cells and moderately in puPAR transfected cells (Figure 4K and Figure 4L). These results suggest that shRNA against uPA, uPAR and U2 inhibit AKT, ERK and NF- κB pathways. These results also show that shRNA against uPA down regulates NICD and HES-induced Notch1, AKT, NF-κB and ERK activation in glioblastoma cell lines.

### Transcriptional inactivation of uPA/uPAR inhibits trafficking of Notch 1 receptor in the U251 MG cell line

**Figure 5:**
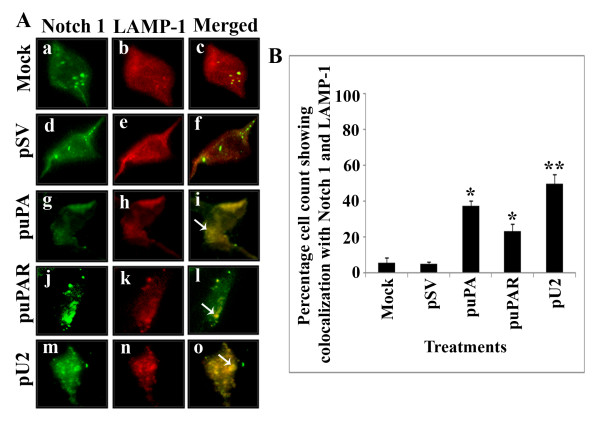
**puPA, puPAR and pU2 inhibits trafficking of Notch 1 receptor in U251 MG cells**. (A) U251 MG cells left untreated (panels a to c) or transfected with pSV (panels d to f), pupa (panels g to i), puPAR (panels j to l) and pU2 (panels m to o) were washed, fixed and permeabilized, and then blocked with normal goat serum and incubated with primary antibodies against Notch 1 and LAMP-1 for 1 hr at RT. The cells were then washed and incubated with Alexa fluor 488 and Alexa fluor 594 conjugated appropriate secondary antibodies. The cells were then washed, and mounted with an anti-fade reagent and viewed under an Olympus fluoview fluorescent microscope. Arrows indicate areas of co-localization. (B) Quantitative representation showing percentage cell count with co-localization of Notch 1 and LAMP1. Data represents average of three independent experiments (**P < 0.01, *P < 0.05; in comparison with the controls).

The trafficking of Notch receptor is known to be mediated by endosomes. To determine if Notch 1 is targeted to lysosomes after transfection with shRNA against uPA, uPAR and U2 (bicistronic construct), we conducted immunofluorescent staining in U251 cells after transfection and checked for co-localization of NICD with LAMP-1 (a lysosomal marker). We did not observe co-localization of Notch 1 with LAMP-1 in mock and pSV-treated cells (Figure 5A, panel a to f) but there were few spots of co-localization in puPA- (Figure 5A, panel g to i) and puPAR- (Figure 5A, panel j to l) treated cells. In pU2-treated cells, we were able to observe a significant association of Notch 1 receptor with LAMP-1, as indicated by a significant number of yellow spots which is suggestive of co-localization (Figure 5A, panel m to o). The percent of cell count showing co-localization is depicted quantitatively in Figure 5B. This result suggests that Notch 1 receptor trafficking is significantly affected in puPA- puPAR- and pU2-transfected U251 MG cells.

### puPA and pU2 affect growth and pre-established intracranial tumors in nude mice

**Figure 6:**
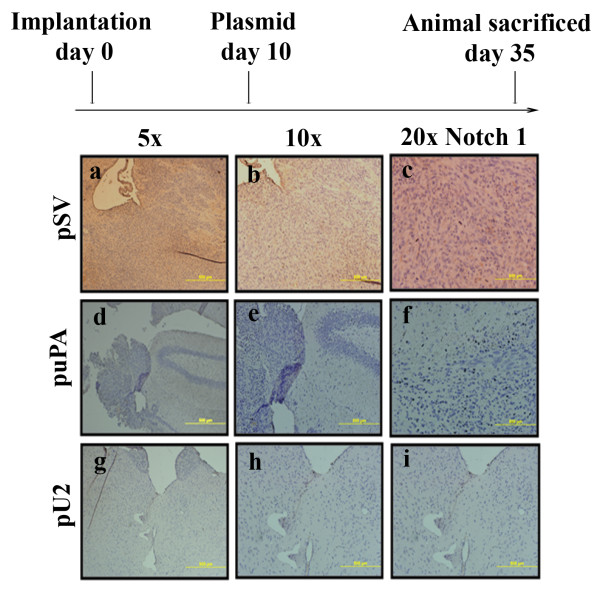
**puPA, puPAR and pU2 inhibits Notch 1 expression in pre established intracranial tumors in nude mice**. (A) U251 MG cells in suspension (2 × 10^6 ^in 10 μl serum-free medium) were injected by the intracranial route. One week later, the mice were injected with either pSV or shRNA-expressing vectors (puPA and pU2) using an Alzet mini osmotic pump (constructs diluted to 1.5 μg ml^-1 ^in PBS and injected at 0.25 μg hour^-1^, with six mice in each group). After a 5-week follow-up period, mice were sacrificed, their brains removed, paraffin embedded and sectioned. Sections were observed and immunohistochemical analysis for Notch 1 was done on paraffin-embedded brain tumor sections of mice treated with pSV, puPA and pU2 with specific antibody for Notch 1.

To further confirm our *in vitro *results, we checked if shRNA against puPA and pU2 affected pre-established intracranial tumors in nude mice and also assessed Notch 1 expression. In U251 MG pre-established intracranial tumors, we observed expression of Notch 1 (Figure 6A, panel a to c), but in puPA-treated tumors, we observed a significant decrease in the tumor margin and infiltration, as well as in inhibition of Notch 1 expression (Figure 6A, panel d to f). In pU2-treated tumors, the tumor margin showed more inhibition than in puPA-treated tumors, and there was also significant inhibition of Notch 1 expression and more inhibition at the tumor margin than in puPA- treated tumors. (Figure 6A, panel g to i). Overall, these results show that uPA and uPAR down regulation in combination inhibits invasion in glioblastoma cells by down regulation of Notch 1 receptor activation and expression, as well as by blocking the trafficking of the Notch 1 receptor.

Expression of Notch 1 has been reported in various types of human cancers [27,28]. Notch 1 has shown to be involved in several functions in cancer cells including proliferation, invasion and angiogenesis [21,29,30]. In this study, we report that Notch 1 is over expressed in glioblastoma cell line U251 MG and xenografts consistent with previous studies [11,12,31]. Studies by us and others have previously shown that simultaneous down regulation of uPA and uPAR in glioblastoma cells significantly down regulated invasion and angiogenesis by *in vitro *and *in vivo *[32,33]. In this study, we found that Notch 1 is a direct target for puPA and by pU2. Studies by many groups [11,34,35] have shown that Notch 1 is target for many genes in glioblastoma, and siRNA against Notch 1 down regulates uPA and inhibits prostate cancer and pancreatic cancer cell invasion [27,36]. Inspite of this current knowledge, the role of uPA and uPAR down regulation in glioma cell invasion remains poorly understood. In this study, we show that knockdown of uPA and uPAR in tandem inhibits cleavage of the Notch receptor, down regulates Notch 1 gene expression and Notch 1 signaling cross talk, and significantly blocks the trafficking of the Notch receptor in glioblastoma cells.

As previously shown in glioblastoma cell line SNB19, we observed that targeted down regulation of uPA and uPAR by single and bicistronic constructs inhibited invasion in U251 MG and in glioma xenografts 4910 and 5310 by Matrigel invasion assay (Figure 1F) and also anchorage independent growth by soft agar colony formation assay (Figure 1D). Increased expression of Notch 1 has been detected in brain tumors [37] and other cancers [38,39]. The role of Notch 1 in different cancers is considerably varied [40]. As previously reported [11], we observed increased expression of Notch 1 in glioma human tissue array, western blot and RT-PCR.

The down regulation of uPA and uPAR inhibited activated Notch 1 activity and cleavage of Notch 1 at the Val 1744 position which resulted in the inhibition of Notch gene transcription. Moreover, glioblastoma cells treated with siRNA against Notch showed decreased uPA expression as shown by western blotting and decreased uPA activity by fibrin zymography. Based on these observations, it is tempting to speculate that the uPA/uPAR system might play a direct role in Notch 1 signaling and that uPA alone could directly regulate Notch 1 in glioblastoma. Whether uPA/uPAR regulates Notch 1 promoter activity is to be studied in future. Our data corresponds to a previous study [41] which showed that Notch 1 knockdown regulates the expression of uPA and uPAR by a distinct gene expression mechanism in prostrate cancer cells. Bin Hafeez et al. [41] conclude their study by stating that in addition to MMP-9, uPA and uPAR might work synergistically to enhance tumor invasion and metastasis in prostrate cancer. As our results show that Notch 1 expression is severely altered with puPA, puPAR and pU2 treatments, we hypothesize that uPA/uPAR could be a target for intervention in glioma.

Previous studies have shown that Notch 1 regulates the PI3K/AKT/mTOR pathway in other cancers. Notch 1 has been reported to cross talk with the AKT pathway in human cancer cells [22-24,42]. Studies by us have also shown that uPA and uPAR down regulation inhibits phosphorylation of Akt [32] in glioblastoma cells. Our results suggest that inhibition of AKT/mTOR signaling pathway via uPA/uPAR down regulation might be mediated by Notch 1 signaling in glioblastoma cells. Phosphorylation of AKT induced by NICD and HES 1 over expression was down regulated by shRNA against uPA in GBM cells. These results suggest the existence of a reciprocal regulation of Notch-1 and AKT pathway in uPA/uPAR down regulation in glioblastoma cells.

Notch is known to cross talk with NF-κB and ERK pathways which are downstream of AKT [27,36]. Our studies have shown that uPA/uPAR down regulation inhibits nuclear activation of NF-κB [43] and phosphorylation of ERK [41]. The present study has also shown that NF-κB and AKT induced by NICD and HES1 over expression in glioblastoma cell lines is inhibited by shRNA against uPA. Further in-depth mechanistic studies are required to understand the role of uPA in regulation of Notch 1-induced AKT, NF-κB and ERK pathways. Our results have comprehensively suggested that uPA/uPAR down regulation results in the inhibition of invasion of GBM cells by targeting the Notch 1 receptor signaling, cleavage, decreased Notch1 mRNA transcription and trafficking. Purow et al [11] have shown that Notch siRNA leads to increased cell death, decreased proliferation and inhibition of cell cycle in glioblastoma cells. Our results suggest that uPA/uPAR down regulation might regulate Notch 1-mediated events in glioblastoma. Interestingly, our results show that uPA/uPAR down regulation resulted in Delta and Jagged downregulation (data not shown). Studies by Purow et al [11] have also shown that Notch ligands could themselves have a potential for transformation and play important roles in gliomagenesis. How shRNA against uPA and uPAR regulates Delta and Jagged is under further investigation. Our results also show that in uPAR antisense cells and in U251 MG cells, uPA was able to activate Notch 1 in the absence of Delta and Jagged ligands. These results suggest that uPA by itself could mediate uPAR independent functions such as Notch 1 regulation in glioblastoma cell lines.

Our studies also indicate that uPA/uPAR down regulation also results in the lysosomal targeting of the Notch 1 receptor in U251 MG cells. The mechanistic details of lysosomal targeting are yet to be explored. Notch 1 expression was also inhibited in pupa- and pU2-treated pre-established intracranial tumors in nude mice. Involvement of uPA/uPAR in a plethora of cellular processes in glioma gives us the advantage of targeting this multifunctional system for therapeutic intervention in patients with high grade glioma.

## Conclusions

In summary, we have presented experimental evidence that strongly suggests the role of Notch 1 in uPA/uPAR down regulation in glioblastoma. shRNA against uPA and uPAR inhibited Notch 1 cleavage, Notch gene transcription and Notch 1-mediated AKT/ERK phosphorylation and NF-κB subunits. From our results, we can conclude that down regulation of uPA/uPAR, singly and in tandem, could be an effective therapeutic approach for inactivation of Notch-1 cleavage, signaling and trafficking and to down regulate Notch-signaling-induced NF-κB, ERK and AKT pathways which are known to play roles in growth, migration, invasion and angiogenesis of glioblastoma. Based on our results, we provide a hypothetical pathway by which uPA/uPAR inhibits the Notch 1 pathway which may be partly mediated by AKT/NF-κB and ERK pathways. Overall, our results suggest that targeting uPA/uPAR may be advantageous for future treatment of gliomas.

## List of Abbreviations

uPA: urokinase plasminogen activator; uPAR: urokinase plasminogen activator receptor; AKT: RAC-alpha serine/threonine-protein kinase; PI3-K: phosphotidyl inositide 3 kinase; ERK:Extracellular regulated kinase; NICD: Notch intracellular domain: HES: Hairy enhancer of split; GAPDH: glyceraldehydephosphate 3 dehydrogenase; LAMP-1: lysosome associated membrane protein 1.

## Competing interests

The authors declare there are no conflicts of interest regarding this manuscript.

## Funding

This research was supported by a grant from National Institutes of Health, CA75557 (to J.S.R.). The contents are solely the responsibility of the authors and do not necessarily represent the official views of NIH. The funders had no role in study design, data collection and analysis, decision to publish, or preparation of the manuscript.

## Authors' contributions

HR conceived and carried out the experiments. HR wrote the manuscript. HR, CG and JSR reviewed and analyzed the data. JSR, MG and DD contributed reagents/materials/analysis tools. All members read and approved the final manuscript.
